# Endoscopic endonasal surgery for optic nerve sheath and optic canal meningiomas: 2D operative video

**DOI:** 10.3171/2025.10.FOCVID25196

**Published:** 2026-01-01

**Authors:** Erik Burgos-Sosa, Yuanzhi Xu, Jonathan Lamano, Vera Vigo, Matei A. Banu, Michael Chang, Collin Liu, Juan C. Fernandez-Miranda

**Affiliations:** Departments of 1 Neurosurgery and; 2 Otolaryngology, Head & Neck Surgery, Stanford University Hospital, Stanford, California

**Keywords:** endoscopic endonasal approach, optic canal meningioma, optic nerve sheath meningioma, supraoptic recess

## Abstract

Meningiomas invading the ventromedial optic canal are uncommon anterior cranial fossa tumors that present with progressive visual decline. Their close relationship with the optic nerve and ophthalmic artery makes surgical intervention highly complex. This video presents two illustrative cases—an optic nerve sheath meningioma and an optic canal meningioma—both treated using an endoscopic endonasal approach (EEA). This direct ventral corridor provides early optic canal decompression and panoramic visualization of critical neurovascular structures, avoiding optic nerve manipulation. The same technique was employed in both cases, but was tailored to the distinct surgical challenges of optic nerve sheath and optic canal meningiomas.

The video can be found here: https://stream.cadmore.media/r10.3171/2025.10.FOCVID25196

## Transcript

### 0:20 Rationale for Procedure.

In this video, we present the endoscopic endonasal approach for meningiomas invading the ventromedial optic canal.

Optic nerve sheath and optic canal meningiomas can affect vision early in their course, making any intervention a balance between tumor control and visual preservation.^1,2^

Several considerations exist when considering microsurgical open approaches, endoscopic endonasal approaches, as well as radiotherapeutic approaches.^3–6^

### 0:45 First Patient: Clinical Presentation and Imaging.

The first case involves a 40-year-old male who presented with a progressive visual deficit in the left eye. Imaging demonstrated a tumor involving the optic nerve sheath with compression of the optic apparatus. An endoscopic endonasal approach was utilized to maximize optic nerve decompression.

### 1:01 Anatomical Essentials.

Some of the key anatomical landmarks are described here, including the superior intercavernous sinus, the lateral opticocarotid recess, and the supraoptic recess.

In addition, the limbus sphenoidale, in continuation with the falciform ligament, as well as the distal dural ring and diaphragma sellae, are demonstrated, the removal of which allows the exposure of the cisternal and intradural segments of the ophthalmic artery.^1^

### 1:24 Surgical Setup and Procedure.

Here, we describe the key points of surgical positioning and preparation.

From an ENT perspective, the nasoseptal flap is harvested up front, accompanied with posterior ethmoidectomies and a wide exposure of the sphenoid sinus, particularly on the left side.

Sellar exposure is done with identification of bilateral carotid prominences, lateral opticocarotid recesses, as well as the anatomical location of the intercavernous sinus. Drilling was then continued superiorly, involving the tuberculum sellae, prechiasmatic sulcus, as well as the planum sphenoidale. Utilizing a Kerrison rongeur and outfracturing technique, the clinoidal segments of bilateral carotid arteries were exposed. Extensive exposure on the left side was necessary to identify and outfracture the optic canal. Anatomical landmarks, including the supraoptic recess and lateral opticocarotid recess, guide this exposure.

Careful drilling of the supraoptic recess was then performed under continuous irrigation. Remaining fragments of the sphenoid wing in the supraoptic recess are removed in order to facilitate 270° decompression of the optic canal.

Further decompression is achieved through drilling and fracturing of the optic strut, all while avoiding injury to the adjacent clinoidal segment of the carotid artery.

At this point, the dura was opened from the level of the prechiasmatic sulcus to the limbus sphenoidale in order to facilitate arachnoid dissection in a right-to-left manner from normal to abnormal anatomy.

Extracapsular tumor dissection is performed carefully, with preservation of the superior hypophyseal arteries, optic chiasm, pituitary stalk, and anterior cerebral artery. In this manner, the tumor can be detached and rotated away from the involved optic nerve. The dura on the left side is further transected in order to facilitate continued extracapsular dissection. This dissection was continued anteriorly in order to expose the optic nerve at the level of the optic canal. Using the right-angle knife, we are able to safely open the falciform ligament and dura of the medial optic canal.

At this point, careful dissection is done to identify the cisternal and intradural segments of the ophthalmic artery by carrying the dissection to the level of the distal dural ring. Using bipolar cautery and microscissors, the tumor was then removed.

The right-angle knife was then used to continue opening the dura of the medial optic canal and further decompress the optic nerve.

Indocyanine green video angiography was then performed to visualize continued vascular perfusion. Here, we confirm preservation of the cisternal and intradural segments of the ophthalmic artery and wide decompression of the optic nerve.

Hemostasis was achieved, and a small piece of Gelfoam soaked in nicardipine was applied onto the optic nerve to reduce the risk of vasospasm of the microvasculature supplying the optic nerve itself.

Skull base reconstruction was performed with a DuraGen inlay, fascia lata autograft, and nasoseptal flap.

### 4:43 Postoperative Course.

This patient had an excellent outcome with resection of the compressive lesion and decompression of the optic canal. Most importantly, the patient experienced visual improvement postoperatively.

### 4:54 Second Patient: Clinical Presentation and Imaging.

The second case involved a 52-year-old female who also presented with progressive visual loss affecting the right eye. Notably, the patient’s right optic nerve was significantly atrophied as a result of compression. MRI demonstrated a tumor invading the right optic canal with superolateral displacement of the optic nerve.

Similar to the first case, an endoscopic endonasal approach was selected.

### 5:19 Surgical Setup and Procedure.

Surgical positioning and preoperative considerations were similar, with a plan for an upfront nasoseptal flap, posterior ethmoidectomies, and an expanded sphenoidotomy favoring the right side.

Intraoperatively, the key anatomical landmarks of the sella were identified: including the optic nerve prominences, carotid prominences, and the lateral opticocarotid recesses. Drilling was performed and again extended superiorly to encompass the tuberculum sellae, prechiasmatic sulcus, and posterior aspect of the planum sphenoidale. Lateral extension of the sphenoidotomy was performed to establish access to the supraoptic recess. In addition, skeletonization of the clinoidal segment of the internal carotid artery and exposure of the anterior wall of the right cavernous sinus was performed to provide generous access to the tumor. As demonstrated previously, drilling was extended into the supraoptic recess to facilitate 270° decompression of the optic nerve.

Drilling was continued to the level of the lateral opticocarotid recess before the sellar dura was opened. This dural incision was then carried superiorly across the superior intercavernous sinus to the level of the prechiasmatic sulcus and limbus sphenoidale. Inferiorly, this dural incision was opened toward the proximal dural ring. At this point, further extension of the dural incision was made superiorly toward the supraoptic recess to promote visualization of the tumor invading the optic canal. The right-angle knife was then used to open the superomedial aspect of the optic canal dura, revealing the tumor entirely within the optic canal.

Careful tumor debulking was then performed using a two-suction technique in order to promote improved visualization of the involved optic nerve. Once appropriately debulked, extracapsular dissection was then performed along available arachnoid planes to separate the tumor from the optic nerve. Further dissection was continued anteriorly, separating the tumor from the optic nerve in a piecemeal fashion. In this case, it became apparent that the tumor was tightly adherent to the optic nerve, so careful dissection with microdissectors was necessary to delineate the plane between the tumor and optic nerve. The right-angle knife was utilized to continue opening the dura of the optic nerve, allowing for further tumor resection and optic nerve decompression. During this portion of the dissection, it was important to anticipate the course of the ophthalmic artery running along the floor of the optic canal to avoid inadvertent injury to this artery. With progressive tumor debulking, the optic nerve was visualized with flattening and pallor consistent with significant compression. Having achieved tumor resection and decompression of the optic nerve, hemostasis was then obtained.

Skull base reconstruction was performed with a DuraGen inlay, followed by placement of an AlloDerm onlay and a nasoseptal flap.

### 9:03 Postoperative Course.

The patient achieved an excellent outcome, and visual improvement postoperatively.

### 9:11 Conclusions.

Overall, the illustrative cases presented here demonstrate the advantages of utilizing the endoscopic endonasal approach for optic nerve sheath and optic canal meningiomas. Particularly, this approach provides direct access to the tumor origin without obstruction or need for manipulation of the optic nerve.

## Disclosures

Dr. Fernandez-Miranda reported personal fees from Medtronic, KLS Martin, and Hotry, and nonfinancial support from Stryker and Storz outside the submitted work.

## Author Contributions

Primary surgeon: Fernandez-Miranda, Banu. Assistant surgeon: Chang, Liu. Editing and drafting the video and abstract: Fernandez-Miranda, Burgos-Sosa, Xu, Lamano, Vigo, Chang, Liu. Critically revising the work: Fernandez-Miranda, Burgos-Sosa, Xu, Vigo, Banu, Chang, Liu. Reviewed submitted version of the work: Fernandez-Miranda, Burgos-Sosa, Xu, Lamano, Banu, Chang, Liu. Approved the final version of the work on behalf of all authors: Fernandez-Miranda. Supervision: Fernandez-Miranda, Burgos-Sosa, Xu, Banu, Chang.

## Supplemental Information

### Patient Informed Consent

The necessary patient informed consent was obtained in this study.
